# Supplementary material for: Obtaining protein foldability information from computational models of AlphaFold2 and RoseTTAFold
Source: Comput Struct Biotechnol J. 2022 Aug 17;20:4481–9. doi: 10.1016/j.csbj.2022.08.034 (PMC9421090; doi:10.1016/j.csbj.2022.08.034)
Supplement: Supplementary data 1 [file mmc1.docx]

**Supplementary Data**

**Obtaining protein foldability information from computational models of AlphaFold2 and RoseTTAFold**

Sen Liu^1,2,3,*^, Kan Wu^1,2,3^, Cheng Chen^1,2,3^

^1^ Key Laboratory of Fermentation Engineering (Ministry of Education), Hubei University of Technology, Wuhan 430068, China

^2^ National "111" Center for Cellular Regulation and Molecular Pharmaceutics, Hubei University of Technology, Wuhan 430068, China

^3^ Hubei Key Laboratory of Industrial Microbiology, Hubei University of Technology, Wuhan, 430068, China

^*^Corresponding author: senliu.ctgu@gmail.com

**Figure S1**. Predicted models of the wild-type DHFRcoli. The X-ray structure (PDB ID: 1RX4) is colored in gray, and the predicted models are colored in spectrum according to the pLDDT values of Cα atoms (red: higher pLDDT values; blue: lower pLDDT values). (a) The top-rank model predicted by AlphaFold2. (b) The lowest-RMSD model predicted by RoseTTAFold. Notice that the spectrums are independently colored in these two predicted models.

**Figure S2**. RoseTTAFold models of DHFRcoli circular permutants with large RMSD values.

**Figure S3**. (a) The matrix of the Spearman r values of the AlphaFold2 models of the foldable DHFRcoli circular permutants. The cells were colored by the Spearman r values shown within them. The experimental data (K_cat_, K_M_, Relative K_cat_/K_M_, deltaG, m-Value) were obtained from {Iwakura:2000ku}. (b) The correlation between the RMSD values of the AlphaFold2 models and the precipitant ratios of the foldable (precipitant ratio < 60%) DHFRcoli alanine insertion mutants. (c) The correlation between the RMSD values of the RoseTTAFold models and the precipitant ratios of the foldable (precipitant ratio < 60%) DHFRcoli alanine insertion mutants.
